# Xylazine as a Drug of Abuse and Its Effects on the Generation of Reactive Species and DNA Damage on Human Umbilical Vein Endothelial Cells

**DOI:** 10.1155/2014/492609

**Published:** 2014-11-11

**Authors:** Luz Silva-Torres, Christian Veléz, Lyvia Álvarez, Beatriz Zayas

**Affiliations:** ^1^Pharmacology and Toxicology Department, School of Medicine, University of Puerto Rico, Medical Science Campus, P.O. Box 335067, San Juan, PR 00936-5067, USA; ^2^Puerto Rico Institute of Forensic Science, PR, USA; ^3^School of Environmental Affairs, Universidad Metropolitana, PR, USA

## Abstract

Human xylazine (XYL) abuse among addicts has received great interest due to its potential toxic effects upon addicts and the need to understand the mechanism of action associated with the potential health effects. XYL is an alpha-2 agonist restricted to veterinarian applications, without human medical applications. Our previous work demonstrated that XYL and its combination with cocaine (COC) and/or 6-monoacetylmorphine (6-MAM) induce cell death through an apoptotic mechanism. The aim of this study was to determine the effect of xylazine on the generation of reactive oxygen species (ROS) and reactive nitrogen species (RNS) as well as DNA damage on endothelial cell. Human umbilical vein endothelial cells (HUVEC) were treated with XYL (60 *μ*M), COC (160 *μ*M), 6-MAM (160 *μ*M), camptothecin (positive control, 50 *μ*M), XYL/COC (50 *μ*M), XYL/6-MAM (50 *μ*M), and XYL/COC/6-MAM (40 *μ*M) for a period of 24 hours. Generation of intracellular ROS, RNS, and DNA fragmentation were analyzed using a fluorometric assay. Results reveal that XYL and 6-MAM increase levels of ROS; no induction of RNS production was observed. The combination of these drugs shows significant increase in DNA fragmentation in G2/M phase, while XYL, COC, and 6-MAM, without combination, present higher DNA fragmentation in G0/G1 phase. These findings support that these drugs and their combination alter important biochemical events aligned with an apoptotic mechanism of action in HUVEC.

## 1. Introduction

Drugs of abuse are habitually adulterated with other substances to enhance or diminish the effects of the final product as well as to increase the value of the drug when sold on the street [1]. Evidence of this adulteration has been reported by the Philadelphia Medical Examiner's Office, indicating detection of XYL and fentanyl, between April 2006 and August 2006, in postmortem samples related to drug intoxication [2]. This combination is highly toxic since both drugs depress the central nervous system. XYL is similar to clonidine in its mechanism of action as alpha-2 receptor agonist, causing bradycardia and transient hypertension followed by hypotension. The blocking of norepinephrine release causes sedation and reduced cardiac output [2, 3]. Meanwhile fentanyl, which is similar to heroin (HER, opioid), in its mechanism of action as *μ* (Mu) receptor agonist, causes sedation, hypotension, and respiratory depression [2, 4, 5]. The pharmacological effects of XYL and HER mixture could induce severe CNS and respiratory depression, considered to be a lethal combination. The combination of HER, XYL, and COC could be inducing addicts to use higher doses because COC produces CNS excitation initially, delaying XYL and HER effects [6, 7]. The use of XYL as heroin adulterant or substitute has increased since 2004 in Puerto Rico and other countries that have reported it [8–13].

The intravenous route is the major route of administration for heroin or XYL drug users, being strongly implicated in human intoxication cases in the United States [2, 3, 10, 12, 14–16]. In these circumstances, it has been suggested that once XYL has gained access into the vascular system, it is distributed within the blood perfusing the target organs such as heart, lungs, liver, and kidney [17]. Upon entering the body, heroin or diacetylmorphine is immediately deacetylated into the blood, producing 6-MAM (6-monoacetylmorphine, heroin metabolite). 6-MAM can cross the blood brain barrier easily and finally is deacetylated to produce MOR, which in turn is the *μ* receptor agonist. During this distribution, the vascular endothelium in contact with XYL could experience toxic effects. Similar distributions are predictable to occur in combination with COC and 6-MAM (heroin metabolite). The endothelial cells are involved in the interchange of metabolites among blood and tissues. In addition to their role in blood homeostasis and wound healing [18, 19], they play an important role in mediating normal physiology and pathophysiology in the human body. Several drugs of abuse, such as XYL, COC, and HER, might be targeting their toxicity to endothelial cells and disturbing their function.

Physiological processes that include vascular tone regulation, mitogenic signaling, and host defense require participation of reactive oxygen and nitrogen species (ROS/RNS) [20]. Nonetheless, disproportionate ROS/RNS production could affect biochemical processes such as cellular signaling, proteins, DNA and could damage lipids, thus interrupting their normal functions. These reactive species (ROS/RNS) include a diversity of relevant species in biological systems, such as superoxide anion, hydrogen peroxide, hydroxyl radical, and peroxynitrite [21–23]. Several of these species are very reactive, while others are more stable. Production of ROS/RNS in cells could be the product of incomplete processes such as enzymatic systems (e.g., NADPH oxidases (NOX1)) and oxygen reduction in the electron transport chain [22, 23]. These reactive species have significant roles in the immune system, participating in several signal transduction pathways, as intra- or extracellular signaling molecules and transcriptional regulation [21]. Non-immune cells such as intestinal epithelial cells also produce ROS/RNS, where considerable amounts of superoxide anion are produced by NOX1, thyroid oxidase (DUOX2), and xanthine oxidase [6]. Therefore sustained high levels of ROS/RNS could trigger severe oxidative stress, generating damage in tissues, which might induce pathologies, such as rheumatic diseases, atherosclerosis, and diabetes [5]. Inflammation and increased ROS levels are also implicated in the pathogenesis of cardiovascular events [24].

Our previous study demonstrated XYL use alone or in combination with COC and/or 6-MAM diminished proliferation and induced apoptosis in EA.hy926 cells (HUVEC) [25]. This apoptosis process could be triggered by increased production of nitrogen and oxygen reactive species. Recent studies also noted that high doses of morphine induced apoptosis are mediated through both the RNS and ROS pathways [26]. The current study investigates the relation of these drugs in the production of reactive species. We intended to determine the presence of DNA fragmentation, ROS and RNS production, in EA.hy926 cells treated with XYL, COC, 6-MAM and their combinations.

## 2. Materials and Methods

### 2.1. Stock Solutions and Reagents

Experimental drugs stock solutions were prepared at concentrations of 3 mM in ethanol 70%, obtained from Sigma Aldrich (St. Louis, MO). Stock solutions were kept in sterile glass vials and stored at 4°C. The positive control, a known apoptosis inducer, camptothecin, cocaine, and xylazine were obtained from Sigma Aldrich; the heroin metabolite (6-monoacetylmorphine) was obtained from Cerilliant Corporation (Round Rock, Texas). The reagent sodium hydroxide (NaOH), 2′,7′-dichlorofluorescein diacetate (DCFH-DA), and 2,3-diaminonaphthalene (DAN) were obtained from Sigma Aldrich (St. Louis, MO). Stock solutions of DCFH-DA were prepared at concentrations of 10 mM in dimethyl sulfoxide (DMSO) biotechnology grade, obtained from Sigma Aldrich, St. Louis, MO. Stock solutions were kept in sterile glass vials and stored at 4°C. DNA fragmentation reagent, 4′,6-diamidino-2-phenylindole (DAPI), was obtained from Chemometec (Allerød, Denmark).

### 2.2. Instrumentation

Countess automated cell counter (Invitrogen, Carlsbad, California) was used for cell quantification. DNA fragmentation was analyzed by cytometry analysis using the NucleoCounter NC-3000 (Chemometec, Allerød, Denmark) instrument. ROS and RNS activation were analyzed using the fluorostar Optima (BMG, Ortenberg, Germany) fluorescence reader.

### 2.3. Cell Culture

The cell line used in this study was human umbilical vein endothelial cell line Ea.hy926, kindly provided by Dr. Cora-Jean S. Edgell, from the University of North Carolina at Chapel Hill (UNC). Cells were cultured on DMEM medium (ATCC, Manassas, Virginia) with 10% fetal bovine serum (ATCC, Manassas, Virginia) [27, 28]. These cultures were maintained at 37°C and 5% CO_2_ [29]. Cell viability was determined with the Countess automated cell counter; once cells reached confluence they were subcultured and treated within 24 hours.

### 2.4. Cell Treatment

The EA.hy926 cells were subcultured and kept at a density of 5.0 × 10^5^ cells per 3.5 ml of culture media plus additives in 25 cm^2^ flasks to assure stable metabolic state and exponential growth. Treatment solutions with the drugs and their combinations were prepared freshly by dilution in medium. Cells were exposed for 24 hours to vehicle (negative control group, 160 *μ*l), and drugs were tested at their approximated IC_50_ (previously calculated [26]): XYL (60 *μ*M), COC (160 *μ*M), 6-MAM (160 *μ*M), camptothecin (positive control group, 50 *μ*M), XYL/COC (50 *μ*M), XYL/6-MAM (50 *μ*M), and XYL/COC/6-MAM (40 *μ*M). 6-MAM was employed instead of HER, because once in the blood stream HER is immediately converted to 6-MAM.

### 2.5. Generation of Reactive Oxygen Species

Determination of ROS generation was performed using 2,7-dichlorofluorescein diacetate (DCFH-DA), as described by Gutiérrez-Praena et al. (2012) and Park et al. (2007) [30, 31]. The production of ROS was assessed after 24-hour treatment in 96-well microplates. When DCFH-DA diffuses across the cell membrane, intracellular esterases hydrolyze it to a nonfluorescent compound (DCFH), which is swiftly oxidized in the ROS presence to extremely fluorescent DCF, proportional to ROS production. After drug treatment, cells were harvested and incubated with 200 *μ*l of 20 *μ*M DCFH-DA in medium at 37°C for 30 min, washed with phosphate buffered saline (PBS), and resuspended in 200 *μ*l of PBS. Cells were then transferred to a 96-well microplate and analyzed. DCFH-DA fluorescent probe converted to DCF [32] reveals reactive oxygen species levels by green fluorescence at an emission wavelength of 535 nm and excitation wavelength of 485 nm using the fluorostar Optima (BMG, Ortenberg, Germany) fluorescence reader. Results (percent, %) were shown as negative control standardization.

### 2.6. Generation of Reactive Nitrogen Species

Generation of RNS was analyzed by implementation of the 2,3-diaminonaphthalene (DAN) performed according to the method by Misko et al. (1993) and Kleinhenz et al. (2003) [33, 34] with minor modifications. In brief, DAN was dissolved in 0.62 N HCl at a concentration of 0.05 mg/ml. After drug treatment, supernatants were recovered and centrifuged at 2,000 g for 2 minutes. Aliquots of these supernatants (85 *μ*l) were placed into 96-well plates (in triplicate), combined with DAN (10 *μ*l) and incubated for 15 min at 37°C. After 15 min, 5 *μ*l of 2.8 N NaOH was added to each well. Samples were analyzed using fluorescence excitation at 360 nm and emission at 440 nm with the fluorostar Optima (BMG, Ortenberg, Germany) fluorescence reader.

### 2.7. DNA Fragmentation Assay

Determination of DNA fragmentation is used as an indicator of apoptosis in drug toxicity assessment. This assay measures cells containing less than 1DNA equivalent (Sub-G1) after degradation of DNA triggered by endonucleases [35]. Cells were harvested after drug treatment and implementing the previously described conditions, fixed with 70% ethanol, incubated 24 hours at 4°C, stained with 500 *μ*l of 1 *μ*g/ml DAPI and incubated for 5 minutes, according to manufacturer's specifications, and analyzed by image analysis with NucleoCounter NC-3000 instrument.

### 2.8. Statistical Analysis

To assess significance in the observed changes in cells exposed to the tested drugs, a one-way ANOVA with Tukey post hoc test was performed, where *P* < 0.05 was considered to be significant. This statistical analysis was performed with Graph Pad Prism software (version 5.03). Results were presented as mean ± SD of 6–9 replicates, from 2 to 3 experiments.

## 3. Results

### 3.1. Results of the Oxidative Stress Assays

**Figure 1:**
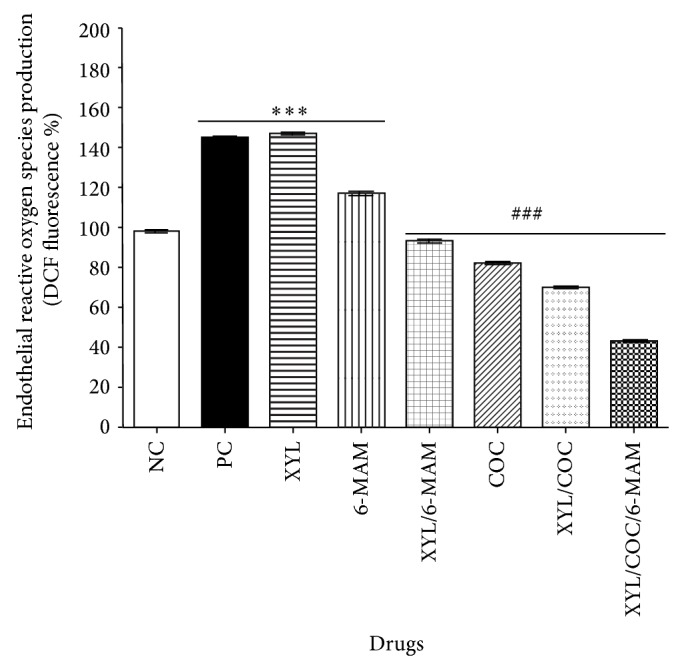
Effect of xylazine, cocaine, 6-monoacetylmorphine, and their combination on ROS production in human umbilical vein endothelial cells (EA.hy926). ROS levels in EA.hy926 cells, after 24 h of exposure to negative control (vehicle), xylazine (XYL, 60 *μ*M), cocaine (COC, 160 *μ*M), 6-monoacetylmorphine (6-MAM, 160 *μ*M), camptothecin (positive control, 50 *μ*M), XYL/COC (50 *μ*M), XYL/6-MAM (50 *μ*M), and XYL/COC/6-MAM (40 *μ*M), were determined. Cells exposed to XYL and 6-MAM showed increased ROS levels. Cells exposed to COC and XYL in combination with COC and 6-MAM showed diminished ROS levels in comparison to negative control as determined by ANOVA. The experiment was repeated for at least three times in replicates and expressed as change from negative control (%). All values are expressed as mean ± SD of 6–9 replicates, from 2 to 3 experiments. Statistical analysis performed was a one-way ANOVA, with Tukey post hoc test, where *P* < 0.05 was considered to be significant. *P* summary; ^***^ significantly higher in contrast to the negative control (*P* < 0.0001), ^###^ significantly lower in contrast to the negative control (*P* < 0.0001).

When EA.hy926 cells were exposed to XYL and 6-MAM at concentrations described formerly, ROS content was significantly enhanced (Figure 1), when compared with the control group. Additionally the ROS content in XYL treated cultures compared to the positive control (camptothecin) was significantly enhanced. Camptothecin is a known apoptosis inducer, topoisomerase I and II inhibitor, DNA intercalating agent, and oxidative stress inducer [36–42]. In contrast cultures treated with COC presented reduced generation of ROS in comparison to the positive control. Furthermore the EA.hy926 cells, exposed to XYL in combination with COC and/or 6-MAM, showed ROS content significantly decreased, when compared to negative control group (Figure 1).

### 3.2. Results of the RNS Determination Assay

**Figure 2:**
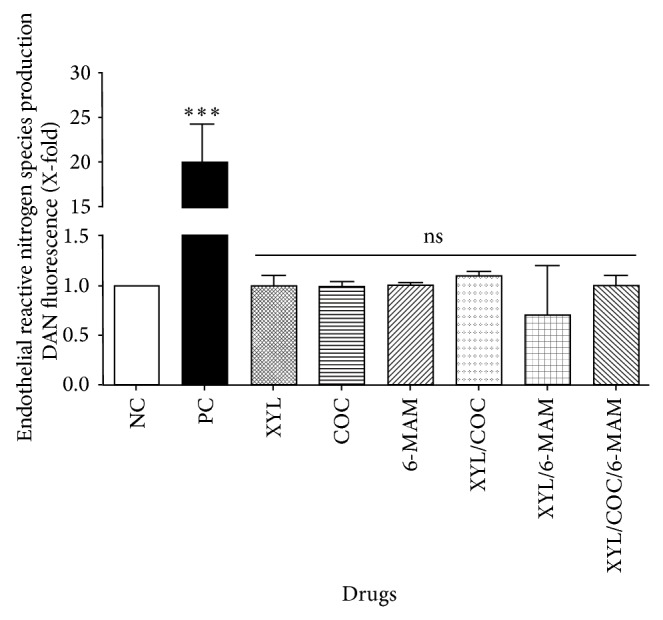
Effect of xylazine, cocaine, 6-MAM, and their combination on RNS production in human umbilical vein endothelial cells (EA.hy926). DAN assay was performed as described previously in the methods section [33, 34]. RNS production after 24 h of exposure to vehicle (negative control), xylazine (XYL, 60 *μ*M), cocaine (COC, 160 *μ*M), 6-monoacetylmorphine (6-MAM, 160 *μ*M), camptothecin (positive control, 50 *μ*M), XYL/COC (50 *μ*M), XYL/6-MAM (50 *μ*M), and XYL/COC/6-MAM (40 *μ*M) was determined. Cells exposed to XYL, COC, 6-MAM, and their combinations exhibited no induction of reactive nitrogen species (RNS) in EA.hy926 cells when compared to the negative control; catalytic activity coincides with basal level production of nitrite and nitrate species. Experiments were repeated for at least three times in replicates. All values are expressed as mean ± SD of 6–9 replicates, from 2 to 3 experiments. Statistical analysis performed was one-way ANOVA, with Tukey post hoc test, where *P* < 0.05 was considered to be significant. *P* summary; ^***^ significantly different from negative control group (*P* < 0.0001), ns = no significant difference from negative control group (*P* > 0.05). Results percent (%) was calculated by negative control normalization.

Assessment of the effect of the tested drugs on total RNS production, which include nitrite and nitrate species, was performed using DAN assay [34]. Results indicate that EA.hy926 cells exposed to XYL, COC, and 6-MAM and their combinations showed no significant generation of RNS (Figure 2), when compared with the negative control. Clearly RNS content was significantly lower when compared to the positive control (camptothecin). Since no significant changes were detected in the RNS production, quantification of nitrate and nitrite released by endothelial cells was not performed.

### 3.3. Results of DNA Fragmentation Assay

**Figure 3:**
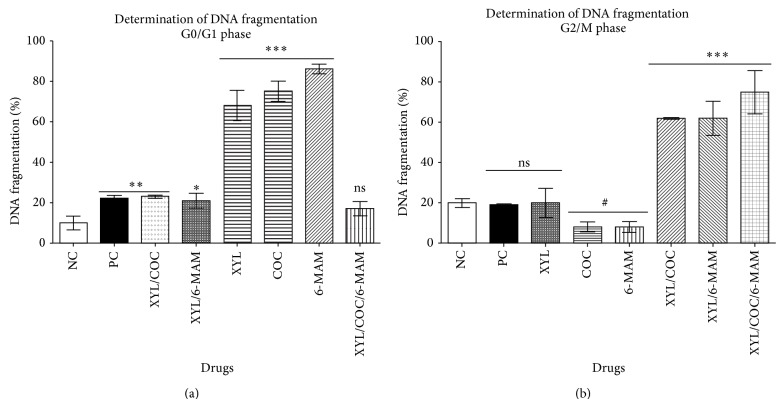
Effect of xylazine, cocaine, 6-monoacetylmorphine, and their combination on DNA fragmentation content in human umbilical vein endothelial cells (EA.hy926). DNA fragmentation content in EA.hy926 cells after 24 h of exposure to vehicle (negative control group, NC), camptothecin (positive control group, PC, 50 *μ*M), xylazine (XYL, 60 *μ*M), cocaine (COC, 160 *μ*M), 6-monoacetylmorphine (6-MAM, 160 *μ*M), XYL/COC (50 *μ*M), XYL/6-MAM (50 *μ*M), and XYL/COC/6-MAM (40 *μ*M) was evaluated in each phase independently and compared to negative control group. DNA fragmentation content assay was performed to measure cells containing less than 1DNA equivalent (Sub-G1) after degradation of DNA triggered by endonucleases or drug interaction [35]. Cells were harvested after drug treatment, fixed with 70% ethanol, incubated 24 hours at 4°C, stained with 1 *μ*g/ml DAPI (5 min incubation), and image-analyzed with NucleoCounter NC-3000 instrument. Results obtained in* phase G0/G1* (a) show significant difference (*P* < 0.0001) in cells exposed to XYL, COC, and 6-MAM. Cells treated with XYL in combination with COC or 6-MAM presented significant difference (*P* < 0.001,  0.01, respectively), but lower than previously described.* Phase G2/M* (b) presented no significant difference (*P* > 0.05, ns) in cells treated with XYL. Cells treated with these three-drug combination presented significant difference (*P* < 0.0001) when compared with the negative control group. The experiment was repeated for at least three times. All values are expressed as mean ± SD of 6–9 replicates, from 2 to 3 experiments. Statistical analysis performed was one-way ANOVA, with Tukey post hoc test, where *P* < 0.05 was considered significant. *P* summary; ^***^ *P* < 0.0001 significantly different when compared to negative control group, ^**^ *P* < 0.001, ^*^ *P* < 0.01, ns = no significant difference from negative control group (*P* > 0.05). Results percent (%) was calculated by negative control normalization.

When EA.hy926 cells were exposed to XYL, COC, and 6-MAM and their combinations at concentrations described previously, differences in DNA fragmentation content among phases G0/G1, S, and G2/M were observed. Phase G0/G1 shows DNA fragmentation with significant difference (*P* < 0.001) in cells exposed to XYL combination with COC. Cells exposed to XYL, COC and 6-MAM presented higher significant difference (*P* < 0.0001). Cells treated with combination of XYL and 6-MAM presented significant DNA fragmentation levels (*P* < 0.01) as well. Cells treated with combination of XYL and 6-MAM presented significant DNA fragmentation levels (*P* < 0.01) as well. Cells treated with combination of the three drugs presented no significant difference (ns, *P* > 0.05) (Figures 3(a) and 3(b)). Cells treated with XYL presented no significant difference in phase G2/M (*P* > 0.05, ns) in DNA fragmentation. Meanwhile cells treated with the three-drug combination presented significant difference (*P* < 0.0001) in DNA fragmentation when compared with the negative control group.

## 4. Discussion

Our study reveals the effect of XYL, COC, 6-MAM, and their combinations on the oxidative species generation of treated EA.hy926 cells. Exposure to XYL and 6-MAM at the experimental concentrations clearly indicated the production of ROS was significantly higher than the negative control and comparable to the positive control (camptothecin). Morphine (MOR) can also induces the production of ROS and apoptosis through increasing intracellular oxidative stress in hepatocytes, macrophages, and glomerular mesangial cells [43–48]. The apoptosis mechanism by ROS characteristically involves receptor activation, mitochondrial dysfunction, and consequently caspase activation by proteins such as the Bcl-2 family. Prior study shows HUVEC treated with MOR presented loss of mitochondrial membrane potential (MMP), in the triggered apoptotic process [49]. Augmented generation of superoxide anions by MOR at a high dose, impairing endothelial function, was also identified. The ROS production has been broadly acknowledged as a significant influence accountable for endothelial dysfunction [50]. This condition has been related to pathologies such as hypertension, diabetes mellitus, vasculitis, aging, systemic, and skin lesions [17, 51–53]. Barrier function is also affected by ROS and RNS, which facilitate increase of endothelial monolayer permeability [54].

This work reveals that the combination of XYL with COC and/or 6-MAM has an antioxidant effect in EA.hy926, since cells treated with this drug combination presented diminished ROS formation in comparison to the negative control. The reduction in oxidative stress in the presence of these drug combinations suggests that these molecules may have a “scavenger” effect once in combination (capacity to neutralize free radicals). Previous studies demonstrated that the heroin, MOR, COC, and their combination inhibit lipid peroxidation in mitochondria isolated from rat brain, presenting the “scavenger” effect [55]. This scavenger effect may be due to inhibition of NADPH oxidase and CYP450, one of the greatest sources of free radicals along with lipoxygenases [56]. Our results are in agreement with this previous study.

Additionally this study has demonstrated that XYL, COC, 6-MAM, and their combination had no effect on the generation of RNS production or nitric oxide (NO) release, which means that the basal level of RNS in EA.hy926 was not disturbed. Studies related to COC effects have reported significant decrease of NO release from bovine coronary artery endothelial cells (BCAECs) [57]; nonetheless this effect was not observed in this study. Previous studies regarding morphine effects have determined that it induces NO release in HUVEC at high doses such as 10^5^ nM (1 mM) [20, 21]. Their results show increased NO production and then decreased MMP in HUVEC treated with MOR. Morphine (at this dose) stimulated NO generation, leading to MMP reduction and consequently apoptosis. Our study used a lower concentration treatment of 6-MAM (precursor of morphine as metabolite) 160 *μ*M (0.16 mM), and induction of RNS production was not observed. Literature review shows that phenothiazine or XYL has not been implicated in the induction of RNS production in EA.hy926 cells, macrophages, or monocytes [58]. The absence of induction in RNS production is indicative of the fact that another pathway could be involved in EA.hy926 cells death when treated with XYL, COC, 6-MAM, and their combination.

DNA fragmentation was measured and correlated to each cell cycle phase. Cells enter the cell cycle in G1 phase, followed by DNA replication in S phase [59]. During G2 phase, two processes happen: DNA repair and preparation for mitosis (M). After M phase, two routes are available; cells could enter G1 or G0 (inactive phase). Cell cycle checkpoints carefully regulate entry into each phase of the cell cycle [46, 47]. DNA damage checkpoints in the cell cycle happen late in G1 phase, which inhibits entrance to S phase, and late in G2, which avoids passage to mitosis. A family of protein kinases regulates the checkpoint control system. DNA damage is known to incite a downstream effect in which the depolarization of mitochondrial membrane and activation of effector caspases are the most probable events suggesting an apoptotic pathway for cell death [59, 60]. DNA fragmentation in G2/M phase has been linked to apoptosis, DNA adduct formation, cyclin dependent kinase (Cdk), and protein phosphatase Cdc25C (proto-oncogene) inhibition [59–62]. Several investigations have proved that DNA-damaging agents such as DNA topoisomerase I and II inhibitors, alkylating compounds, or irradiation could trigger the inhibition of p34cdc2 kinase activity. One of these agents is doxorubicin (DOX), whose mechanism of action produces arrest at G2/M phase, increased frequency of DNA damage, and inhibition of p34cdc2 kinase. Also it has been discovered that DNA double strand breakage and inhibition of p34ı2 kinase activity, caused by DOX, are correlated [61].

This is consistent with the observed effects on EA.hy926 cells treated with XYL, COC, and 6-MAM and XYL combination with COC, showing significant DNA fragmentation in G0/G1 phase. Meanwhile cells treated with the combination of these drugs presented significant DNA fragmentation in G2/M phase. DNA fragmentation in both phases, G0/G1 and G2/M, are related to the apoptotic process [59, 61, 63–65].

## 5. Conclusion 

This study reports primarily the toxic effects of XYL and its combination with COC and/or 6-MAM on the EA.hy926 cell line, human umbilical vein endothelial cells. Our results demonstrate that the effects of XYL and 6-MAM are similar to those induced by camptothecin (positive control), when their ROS induction was compared. The effects of the drug combination are similar to those induced by COC alone, acting as scavengers. Regarding induction of RNS production, all drugs and their combination showed no effect. Meanwhile analyses of the DNA fragmentation effects were observed in two groups (cell cycle phases). Cells treated with XYL, COC, 6-MAM (*P* < 0.0001), and XYL combination with COC (*P* < 0.001) presented higher DNA fragmentation in G0/G1 phase when compared to negative control. Cells treated with combination of XYL and 6-MAM also presented significant difference (*P* < 0.01). Drug combinations presented higher DNA fragmentation in G2/M phase when compared to negative control. These findings indicate that two distinct mechanisms are present, interacting with different cell cycle checkpoints.

In summary, induction of ROS production could be related to DNA fragmentation and the apoptosis process in cells treated with xylazine and 6-monoacetylmorphine, while ROS and RNS production is not related to DNA fragmentation or apoptosis process in cells treated with cocaine and the three-drug combination. This suggests the presence of two mechanisms of action, one in which cocaine is acting as a scavenger but causing DNA damage by a different unknown pathway. Cocaine in combination with XYL and/or 6-MAM enhances the scavenger effect without inhibiting DNA fragmentation. These interactions remain unknown and should be studied in detail. This is the first study of XYL effects upon human endothelial cells due to the use of this substance as a drug of abuse. XYL has only been approved for veterinary applications and thus the effects on human cells are unclear. The lack of reference studies related to XYL effects on humans makes the correlation of results difficult.

Understanding the toxicity mechanisms induced by xylazine and the combination with speedball (cocaine and heroin mixture) on endothelial cells has allowed us to visualize the possible multiple adverse effects. These findings contribute to the understanding of potential health impacts in users of xylazine (alone) and its combination with cocaine and/or 6-monoacetylmorphine (heroin metabolite). Health professionals could develop specific treatment to address this possible particular tissue damage and its consequences. In addition, physicians can be alert to these effects and potentially can recommend which detoxification treatment could be used in this addicts population; to manage possible dysfunction involving highly perfused organs such as liver and kidney. These new insights into the cellular effects of these drugs can allow clinicians to update procedures for dealing with the effects of xylazine and its combinations.
